# School Ethos and Recurring Sickness Absence: A Multilevel Study of Ninth-Grade Students in Stockholm

**DOI:** 10.3390/ijerph17030745

**Published:** 2020-01-23

**Authors:** Sara Brolin Låftman, Joacim Ramberg, Bitte Modin

**Affiliations:** 1 Department of Public Health Sciences, Centre for Health Equity Studies (CHESS), Stockholm University, SE-10691 Stockholm, Sweden; bitte.modin@su.se; 2 Department of Special Education, Stockholm University, SE-10691 Stockholm, Sweden; joacim.ramberg@su.se

**Keywords:** school effectiveness, school ethos, school refusal, students, health, contextual

## Abstract

School absence has been identified as a severe problem in Sweden, both at the individual level and for society as a whole. Despite the multitude and complexity of reasons behind school absence, health-related problems are likely to be one important determinant. This indicates that knowledge about factors that may contribute to preventing health-related absence among students is relevant. The aim was to investigate whether a higher level of teacher-reported school ethos was associated with less recurring sickness absence among students. Data from four cross-sectional surveys performed in 2014 and 2016 were combined. The Stockholm School Survey was carried out among 9482 ninth-grade students (ages 15–16 years) in 150 school units, and the Stockholm Teacher Survey was performed among 2090 teachers in the same units. School ethos was operationalised by an index of 12 teacher-reported items that was aggregated to the school-level. Recurring student sickness absence was captured by self-reports and defined as absence on >10 occasions during the current school year. Two-level logistic regressions were performed. The results show that about 9.5% of the students reported recurring sickness absence. Students attending schools with higher levels of teacher-rated school ethos were less likely to reporting recurring sickness absence than those attending schools with lower levels of ethos, even when adjusting for potential confounders (OR 0.79, 95% CI 0.65–0.97). In conclusion, recurring sickness absence was less common among students attending schools with higher levels of teacher-rated ethos. The findings suggest that schools may contribute to promoting student health.

## 1. Introduction

School absence has been identified as a severe problem in Sweden, both at the individual level and for society as a whole [1]. Students with extensive absence do not receive the education they are entitled to [2], with potentially negative consequences in terms of lower school performance [3] and therefore possibly also poorer future prospects. Even though it is unclear whether school absence has become more common over time, it is likely that recurring absence from school has especially severe consequences in today’s knowledge society [1].

The reasons for school absence are heterogeneous and complex [1]. Yet, a Norwegian study that examined reasons for school nonattendance among students in grades 6–10 showed that students themselves reported subjective health complaints as the most common reason, followed by somatic symptoms, truancy, and school refusal, in that order [4]. A Swedish study on teachers’ views of the causes behind problematic school absenteeism reported that teachers rated family factors as the most important causes (i.e., problematic home-situation and parental permissive style), and thereafter students’ mental health-related problems (i.e., low mood or depression, and nervousness, worry and anxiety) [5]. Hence, despite the multitude and complexity of reasons behind school absence, health-related problems are likely to be one important determinant. Accordingly, knowledge about factors that may contribute to preventing health-related absence among students is relevant. One important setting for such preventative factors is the school itself.

So-called “effective schools” are characterised by high expectations in relation to academic performance, clear goals, continuous feedback, regular follow-up of students’ progress, an orderly environment, and strong relations between parents and the school [6,7]. A strong school ethos is one key feature of effective schools. School ethos refers to the norms, attitudes and behaviours that characterise the social interaction among teachers and students [6,8]. The concept of school climate is closely connected to that of school ethos in that both terms refer to the overall “atmosphere” and social features of the school context. Yet, while school climate tends to be captured by students’ experience of the social school environment [9,10], school ethos refers to properties that have been purposefully imposed from higher levels in the school organisation through the leadership [8]. Studies have shown that a strong school ethos is linked with higher student performance [6,11] but also with less student involvement in health risk behaviors [7,12,13]. Whether a strong school ethos is associated also with less student sickness absence is however less known. Yet, prior studies have shown that a certain portion of the variation in subjective health complaints among students can be attributed to the school-level [14,15], suggesting that shared conditions in school affect students’ subjective health. School ethos is one factor that may influence student health. More specifically, it is reasonable to assume that a strong school ethos promotes subjective health and counteracts milder psychological problems among students. There are however fewer reasons to believe that longstanding illness and chronic disease conditions vary across schools when controlling for student-level characteristics, and it is also unlikely that school ethos may have an impact on this type of health outcomes.

The aim of the current study was to investigate whether higher levels of teacher-reported school ethos were associated with lower levels of recurring sickness absence among students, when adjusting for relevant confounders at the student- and the school-level.

## 2. Materials and Methods

### 2.1. Data Material

The data were derived from four cross-sectional surveys conducted in 2014 and 2016: The Stockholm School Survey (SSS) and the Stockholm Teacher Survey (STS). In addition, school-level official register information from the Swedish National Agency for Education was used. The SSS is performed every second year among students in the ninth grade of compulsory school (ages 15–16 years) and the second grade of upper secondary school (ages 17–18 years) in all public schools and a large number of independent schools in Stockholm municipality. Students complete the questionnaires with pencil and paper in the classroom. The response rate has been estimated to 76% in 2014 and 78% in 2016 [16]. The STS is a web survey that was carried out among teachers in 2014 and 2016. The overarching goal of the STS was to gather information about school-contextual features, including teachers’ ratings of the school ethos as well as other aspects of the school environment and teachers’ working conditions. The response rate among senior-level teachers was 54% in both 2014 and 2016. School-level measures were constructed by using the school mean of teachers’ ratings. These variables were merged with the student-level data from the SSS. In the present study, we used data based on survey information collected among ninth-grade students (15–16 years) in the SSS with linked school-level information collected among senior-level teachers in the STS, as well as register data on schools provided by the Swedish National Agency for Education. Information from all three data sources was available for 150 senior-level schools, including responses from 10,288 students and 2090 teachers. The high number of teachers in relation to students is due to the fact that only students in the ninth grade were surveyed in the SSS, while the STS was sent to all teachers working in grades 7–9 at the same schools. For the analyses, we excluded 806 students with internal nonresponse, rendering a study sample of 9482 students distributed across 150 school units (i.e., 92.2% of the original sample). Further details on the data material are provided in the Technical Report [17].

### 2.2. Ethics

The SSS is completed anonymously by students, with no information on personal identification. Therefore, the data are exempt from ethical approval, according to a decision by the Regional Ethical Review Board of Stockholm (2010/241-31/5). The STS and its linkage to the SSS has been approved ethical permission from the Regional Ethical Review Board of Stockholm (2013/2188-31/5; 2015/1827-31/5).

### 2.3. Measures

#### 2.3.1. Dependent Variable

Recurring sickness absence was measured by the question in the SSS: “Have you been absent from school this year because you were ill or felt poorly?” and the response categories “No”, “Yes, once”, “Yes, 2–3 times”, “Yes, 4–10 times”, “Yes, 11–20 times” and “Yes, more than 20 times”. Students who marked either of the two last options (i.e., >10 times) were classified as having reported recurrent sickness absence.

#### 2.3.2. Independent Variable

*School ethos* was measured by a summative index based on twelve items in the STS: (a) “At this school we have a value system (‘värdegrund’) which is clear to students”; (b) “At this school the teachers make an effort to provide positive feedback about students’ performance”; (c) “Teachers have high expectations of student performance”; (d) “Teachers at this school take their time with students even if they want to discuss something other than school work”; (e) “At this school we actively work on issues such as violence, bullying and harassment among students”; (f) “This school provides a stimulating learning environment”; (g) “The teachers at this school have a strong work ethic”; (h) “The teachers work with strong enthusiasm”; (i) “At this school the students are treated with respect”; (j) “The teachers at this school feel confident as classroom leaders”; (k) “At this school students’ motivation is a stimulating part of work”, and (l) “There is high staff turnover amongst teachers at this school”. The response options were (5) “Strongly agree”; (4) “Agree”; (3) “Neither agree nor disagree”; (2) “Disagree”; and (1) “Strongly disagree”, except for the last item (l) which had a four point scale with the response categories (1) “Agree completely”, (2) “Agree somewhat”, (3) “Disagree somewhat”, and (4) “Disagree completely”. The items were summed to an index with the possible range 12–59. The measure was developed through exploratory and confirmatory factor analysis and demonstrated good psychometric properties (the root square mean error of approximation (RMSEA) = 0.09, the Tucker-Lewis index (TLI) = 0.92, the comparative fit index (CFI) = 0.93) and high internal consistency (Cronbach’s alpha = 0.90) [17]. The mean value of the index for each school unit was utilised as a school-level measure of ethos.

#### 2.3.3. Control Variables (Student-Level)

Gender was captured by the question “Are you a boy or a girl?” with the response categories “Boy” and “Girl”.

Family structure was measured by the question “Which people do you live with?” with a list of boxes to be marked. Students who ticked “Mother” and “Father” were coded as living with two parents in one household and were contrasted against all others.

Parental university education was captured through the question “What is the highest education your parents have?” The response categories (for both mother and father) were: “Old elementary school (folkskola) or compulsory school (max 9 years schooling)”, “Upper secondary school”, “University and university college”, and “Don’t know”. Because large proportions of students reported “Don’t know” or skipped the question, a dichotomous measure was constructed, where students who marked “University and university college” for one or both parents were coded as having at least one parent with university education, and contrasted against all others.

Parental unemployment was captured through the question “What do your parents do?” with a number of response categories for both the mother and the father. Those who marked “Unemployed” for one or both parents were coded as having at least one parent who is unemployed, and were contrasted to all others.

Migration background was assessed by the question “How long have you lived in Sweden?” The response categories were: “All my life”, “10 years or more”, “5–9 years” and “Less than 5 years”. A dichotomous measure was created, with the two first categories contrasted against the two latter.

#### 2.3.4. Control Variables (School-Level)

*School segregation profile* was constructed by means of Latent Class Analysis, using four criteria: (1) parents’ average education; (2) proportion of students born abroad; (3) proportion of recently immigrated students (i.e., within the past 4 years); and (4) students’ average academic motivation. Information about the first three criteria was provided by the Swedish National Agency of Education, and information about the fourth criterion was derived from students’ ratings in the SSS. Four clusters were identified: “privileged”, “typical”, “deprived” and “deprived immigrant” schools. More detailed information on the measure is provided elsewhere [11,17].

*School type* was based on information from the Swedish National Agency of Education and had two categories: public and independent.

### 2.4. Statistical Method

The statistical method was multilevel modelling. Two-level binary logistic regression models were estimated in Stata (version 15) using the “xtmelogit” command. To assess potential non-linear associations between teacher-rated school ethos and recurring sickness absence among students, the school ethos measure was divided into three categories of about equal size, in order to distinguish schools with a relatively weak, intermediate, and strong ethos. In addition, we ran a model with the continuous measure of school ethos, with results reported in the text. For all models, the Intra Class Correlation (ICC) is reported. In multilevel binary logistic regression, this is an approximation of the proportion of the variation in the dependent variable that can be attributed to the higher-level unit in the data.

## 3. Results

Descriptive statistics of the variables are presented in Table 1. According to our operationalization, about 9.5% of the students reported recurring sickness absence.

Next, we examined the prevalence of recurring sickness absence by schools’ level of teacher-rated ethos (Table 2). A gradient pattern was found, with recurring sickness absence being least common among students attending schools with relatively low teacher ratings of the ethos (11.3%), somewhat less common among students in schools with intermediate level of teacher-rated ethos (9.7%), and least common among those attending schools with a relatively strong ethos (7.6%). A chi-square test showed that there was a statistically significant difference between categories (χ^2^ = 24.23, *p* < 0.001).

Subsequently, a series of multilevel models were performed, with results reported in Table 3. The empty model shows that approximately 2.3% of the total variation in recurring sickness absence could be attributed to the school-level. Model 1 added student-level characteristics. Girls were shown to have a greater likelihood than boys of reporting recurring sickness absence (OR 1.69, 95% CI 1.46–1.94). Students not living with two parents in the same household were more likely to report recurring sickness absence compared with those living with two parents (OR 1.62, 95% CI 1.40–1.87). Students with at least one university-educated parent were less likely to report recurring sickness absence compared to those with no university-educated parents (OR 0.69, 95% CI 0.60–0.80). Parental unemployment was associated with an increased risk of recurring sickness absence (OR 1.67, 95% CI 1.31–2.14). There was no statistically significant difference by migration background, when adjusting for the other sociodemographic characteristics (OR 1.16, 95% CI 0.93–1.46). In Model 2, school segregation cluster and school type were added. Compared with student in privileged schools, those attending a typical school were more likely to be recurrently sickness absent (OR 1.48, 95% CI 1.18–1.87) and an even higher likelihood was found for those attending deprived schools (OR 1.92, 95% CI 1.44–2.56). There was however no statistically significant difference between students in privileged and those in deprived immigrant schools. Furthermore, no statistically significant difference by school type was shown (OR 1.03, 95% CI 0.84–1.26). In Model 3, school ethos was added. The analysis showed that students attending schools with a relatively strong teacher-rated ethos were less likely to report recurring sickness absence compared with those in schools with a relatively weak teacher-rated ethos (OR 0.79, 95% CI 0.65–0.97). However, there was no statistically significant difference between students in schools with an intermediate vs. strong level of ethos (OR 0.91, 95% CI 0.77–1.09). We also ran a model including the continuous, z-standardised school ethos variable instead of thirds. This analysis did however not demonstrate any statistically significant association between school ethos and recurring sickness absence among students (OR 0.95, 95% CI 0.88–1.03) (results not presented in table).

## 4. Discussion

This study showed that recurring sickness absence was less common among students attending schools with higher levels of teacher-rated ethos. The study extends previous research which has shown that a strong school ethos is linked with higher academic achievement [6] and less engagement in health risk behaviors among students [7,12,13]. The findings are also in line with those of a previous study based on the same data material as the present one, which demonstrated that higher levels of teacher-rated school ethos were associated with a lower likelihood of truancy at the student level [18]. It should however be emphasised that the amount of variation in recurring sickness absence that could be attributed to the school-level was rather limited, implying that individual-level factors in general are more important. This finding is in line with studies of well-being outcomes, which have shown that a relatively small portion of the variation in student well-being can be accounted for by the school-level [14,15].

A pertinent question is how the association between teachers’ ratings of the school ethos and student sickness absence may be understood. Possible mechanisms include student performance, academic motivation, and strong teacher–student relations. Indeed, earlier studies based on the same data material have shown that teacher-rated school ethos is positively associated with academic achievement [11] as well as perceived teacher caring [19]. It is well-established that health problems, in turn, are inversely associated with academic performance [20] as well as with perceived teacher support [21,22,23,24]. Further, effective schools are characterised not only by strong student–teacher relations but also by strong parent–school relationships, although this was not explicitly captured by our measure of school ethos. Nevertheless, it is possible that also strong parent–school relationships may contribute to understanding the association between school ethos and student sickness absence. Indeed, in a survey on illegitimate school absence performed among principals by the Swedish Schools Inspectorate [2], the respondents emphasised that the school’s relationships with students as well as parents are key components in the endeavour to reduce school absence.

Even though the present study did not focus on the role of individual- and family-level characteristics for recurring sickness absence, the clear sociodemographic differences that were observed merit attention. The results showed that recurring sickness absence was more common among girls than boys, reflecting the well-established empirical pattern that girls report more subjective health complaints than boys [25,26]. Furthermore, not living with two parents in the same household, not having a university-educated parent, and having a parent who is unemployed, were all associated with a higher likelihood of recurring sickness absence, indicating palpable social inequalities in health among youth along these structural dimensions. Also at the school-level, it was shown that students attending schools whose student composition could be characterised as “typical” or (especially) “deprived” were more likely to be recurrently sickness absent compared to students in schools with a “privileged” student composition. Given the possible negative implications of school absence for academic achievement [3] and potentially also for future prospects, the links between both individual-/family- and school-level sociodemographic characteristics and recurring sickness absence are an important topic for further investigation.

Some previous studies have shown a nonexistent association between school absence and sickness absence in adulthood [27,28], although one long-term follow-up study reported that for women but not for men, occasional sickness absence from school at age 16 predicted sickness absence in adulthood [29]. Yet, these studies do not necessarily indicate that measures of school sickness absence in general have low validity. As shown in the study by Havik et al. [4], students themselves reported subjective health complaints as the most common reason for school absence, and as demonstrated by Gren-Landell et al. [5], teachers emphasised students’ mental health problems as one important factor behind problematic absence from school. These forms of health problems are not chronic in character, but are affected by social circumstances. Regarding subjective health complaints, Inchley et al. [25] (p. 79) have phrased it accordingly: “Having multiple health complaints is an important indicator for measuring subjective well-being, as it reflects individual burden and personal experience related to negative life events in the social context of family, school and peers”. In other words, if subjective health complaints and mental health problems are important for school absence, the lack of a clear and consistent association between school absence and adult sickness absence is not surprising.

The study’s main strength is the data material used, with rich information from both students and teachers in a large number of lower secondary school units. The fact that school ethos was reported by teachers and recurring sickness absence by students is an advantage, since this reduces the risk of common methods variance. Furthermore, using official registry data on schools provided excellent opportunities to adjust for potential school-level confounders, thus enabling us to isolate the association between school ethos and student recurring sickness absence as far as possible. There are however also limitations. The fact that recurring sickness absence was based on student self-reports may limit the validity of the measure. A more neutral source would have been preferable. Information on the reasons for sickness absence would also have been highly valuable. From a theoretical point of view, it can be assumed that school ethos may influence students’ subjective health, while there are fewer reasons to expect an association with longstanding illness or chronic disease. The data used in the present study did however not include any information on the reasons behind the students’ sickness absence, and hence this assumption could not be confirmed empirically. It could be assumed that students who played truant were inclined to mark that they had been recurrently sickness absent from school; however, since there was a specific question on truancy in the questionnaire it is likely that the students differentiated between these two types of absence in their responses. Furthermore, despite the relatively high response rate in the Stockholm School Survey, one limitation concerns the fact that students with recurring sickness absence are likely to be systematically underrepresented in the data. This implies that the prevalence of recurring sickness absence may be somewhat underestimated. Yet, we do not see any reasons why such a response bias would affect the associations found. Another limitation concerns the fact that we were not able to fully control for the selection of students into schools. Even though we adjusted for student-level sociodemographic characteristics as well as school segregation profile and school type, it cannot be ruled out that unobserved characteristics may partly account for the associations found (e.g., that students with certain kinds of problems are more likely to report recurring sickness absence and also more likely to attend schools with lower levels of ethos). Finally, the data were collected among teachers and students in lower secondary schools in Stockholm, which may limit generalizability to other populations. To validate the findings, studies of other age groups as well as of other geographical contexts and school systems are needed.

Empirically grounded recommendations on how school ethos may be improved is beyond the scope of this study. Yet, a strong and visionary school leadership is likely to be a prerequisite for a prosperous school ethos. Furthermore, teachers’ work environment can be assumed to play a key role. A work environment characterised by features such as manageable demands, high control, and acceptable amounts of administrative work, is likely to present with greater opportunities for teachers to promote a strong school ethos in terms of, e.g., providing constructive feedback to students, monitoring students’ progress, and maintaining strong relationships with students and parents. A prior study based on the same data material as the current one showed that schools’ levels of ethos varied with the schools’ student composition, in that schools with a more socioeconomically privileged student composition had higher levels of teacher-rated ethos [11]. Thus, strengthening the ethos in schools with a more socioeconomically disadvantaged student composition seems especially important in pursuit of more equal opportunities for all students. Finally, to gain enhanced knowledge about the role of school ethos for student outcomes, a recommendation for future research is to study schools’ levels of ethos over time, and to measure whether changes in teacher-rated ethos at a school are associated with changes in student outcomes. If this is the case, it is also relevant to examine if associations between school ethos and student outcomes are similar across different categories of students, or if some groups of students benefit more (or less) from a strong school ethos.

## 5. Conclusions

Using data collected among teachers and students in Stockholm, this study showed that recurring sickness absence was less common among students attending schools with higher levels of teacher-rated ethos. Accordingly, the findings suggest that strengthening schools’ ethos may contribute to reducing sickness absence among students. Phrased differently, this study establishes that school is not only a setting for learning, but that it may also contribute to promoting student health.

## Figures and Tables

**Table 1:** Descriptive statistics of variables included in the analyses. N = 9482.

*Student Level*	n	%	
Recurring sickness absence	905	9.5	
Gender			
Boy	4757	50.2	
Girl	4725	48.8	
Family structure			
Two parents in the same household	6234	65.8	
Other	3248	34.2	
Parental university education			
No parent/information missing	3981	42.0	
At least one parent	5501	58.0	
Parental unemployment			
No parent	8944	94.3	
At least one parent	538	5.7	
Migration background			
≥10 years in Sweden	8648	91.2	
<10 years in Sweden	834	8.8	
*School Level*	n	%	
School segregation profile			
Privileged	1718	18.1	
Typical	5308	56.0	
Deprived	1085	11.4	
Deprived immigrant	1371	14.5	
School type			
Public	8020	84.6	
Independent	1462	15.4	
	Mean	S.d.	Range
School ethos	46.54	4.20	30.00–57.00
Weak (n = 3166)	42.10	3.25	30.00–45.31
Intermediate (n = 3199)	46.76	0.83	45.33–48.38
Strong (n = 3117)	50.81	1.94	48.43–57.00

**Table 2:** Recurring sickness absence among students, by thirds of teacher-rated school ethos n = 9482.

	n	%	χ^2^
School ethos			
Weak	357	11.3	
Intermediate	310	9.7	
Strong	238	7.6	24.23 ***

*** *p* < 0.001.

**Table 3:** Recurring sickness absence (>10 times vs. less) among students regressed on teacher-rated school ethos. Odds ratios from two-level binary logistic regression models. n = 9482 students distributed across 150 school units.

	Empty Model	Model 1		Model 2		Model 3	
		**OR**	**95% CI**	**OR**	**95% CI**	**OR**	**95% CI**
*Student Level*							
Gender							
Boy (ref.)		1.00	–	1.00	–	1.00	–
Girl		1.69 ***	1.46–1.94	1.70 ***	1.47–1.96	1.70 ***	1.48–1.96
Family structure							
Two parents in the same household (ref.)		1.00	–	1.00	–	1.00	–
Other		1.62 ***	1.40–1.87	1.60***	1.38–1.84	1.59 ***	1.38–1.84
Parental university education							
No parent (ref.)		1.00	–	1.00	–	1.00	–
At least one parent		0.69 ***	0.60–0.80	0.72 ***	0.62–0.84	0.73 ***	0.63–0.84
Parental unemployment							
No parent/information missing (ref.)		1.00	–	1.00	–	1.00	–
At least one parent		1.67 ***	1.31–2.14	1.69 ***	1.33–2.17	1.69 ***	1.32–2.16
Migration background							
≥10 years in Sweden (ref.)		1.00	–	1.00	–	1.00	–
<10 years in Sweden		1.16	0.93–1.46	1.17	0.93–1.48	1.17	0.93–1.48
*School level*							
School segregation profile							
Privileged (ref.)				1.00	–	1.00	–
Typical				1.48 **	1.18–1.87	1.36 *	1.07–1.73
Deprived				1.92 ***	1.44–2.56	1.69 **	1.25–2.28
Deprived immigrant				1.29	0.96–1.72	1.13	0.83–1.54
School type							
Public (ref.)				1.00	–	1.00	–
Independent				1.03	0.84–1.26	1.06	0.87–1.30
School ethos							
Weak (ref.)						1.00	–
Intermediate						0.91	0.77–1.09
Strong						0.79 *	0.65–0.97
ICC	2.3%	1.3%		0.4%		0.1%	

*** *p* < 0.001 ** *p* < 0.01 * *p* < 0.05.
